# The Floras of Southern and Tropical Southeastern Yunnan Have Been Shaped by Divergent Geological Histories

**DOI:** 10.1371/journal.pone.0064213

**Published:** 2013-05-28

**Authors:** Zhu Hua

**Affiliations:** Key Laboratory of Tropical Forest Ecology, Xishuangbanna Tropical Botanical Garden, Chinese Academy of Sciences, Kunming, Yunnan, P. R. China; Wuhan Botanical Garden, Chinese Academy of Sciences, Wuhan, China, China

## Abstract

The southern and tropical southeastern regions of the Yunnan Province in southwestern China have similar monsoonal climates and lowland tropical rain forest vegetations. The floras of both regions are dominated by tropical floristic elements (78.3% in southern Yunnan and 68.83% in southeastern Yunnan), and both belong to the Indo-Malaysian flora at the northern margin of tropical Asia. However, some temperate East Asian characteristic families are well represented in the flora of tropical southeastern Yunnan, while families characteristic of tropical Asia are well represented in the flora of southern Yunnan. Additionally, there are 14 mainly east Asian families in tropical southeastern Yunnan that are not found in southern Yunnan. Although the two regions share 80% of their genera, 237 genera are restricted to southern Yunnan, and 349 genera to tropical southeastern Yunnan. Furthermore, 57 genera with an East Asian distribution, 53 genera with a North temperate distribution, 22 genera endemic to China, and 17 genera with an East Asia and North America disjunct distribution are found only in tropical southeastern Yunnan. The flora of tropical southeastern Yunnan is more closely related to Eastern Asian flora, while the flora of southern Yunnan is more closely related to Indo-Malaysian flora. The divergence of the flora is well supported by the geological history of the region; the flora of tropical southeastern Yunnan was mainly derived from the South China Geoblock, while the southern Yunnan flora derived from the Shan-Thai Geoblock.

## Introduction

Yunnan, in southwestern China, is a region of exceptional interest to biologists not only because it is situated in a transitional zone between tropical south-east Asia and temperate east Asia [1], [2], but also because it was at a sutural zone between Gondwana and Laurasia [3], [4], [5]. The origin and evolution of the Yunnan flora were largely influenced by the uplift of the Himalayas, the formation of the east Asian monsoon climate and the extrusion of the Indochina block into tropical SE Asia since the later Tertiary [6].

Little was known about the tropical flora and vegetation of southwestern China until the late 1950s because of poor access. After a China-Russia expedition in the late 1950s, which penetrated deep into southwestern China, including southern Yunnan, it was suggested that tropical rain forests existed in southwestern China, but these were considered different from those in Indo-Malaysia [7], [8]. Further biogeographical and ecological studies on the vegetation and flora of tropical southwestern China revealed that it is comprised of Indo-Malaysian flora and has true evergreen rain forest with the same forest profile and physiognomic characteristics as equatorial lowland rain forests [9], [10], [11], [12], [13], [14], [15], [16], [17], [18], [19], [20], [21], [22], [23], [24], [25], [26], [27].

The tropical flora in southern Yunnan differs from that of southeastern Yunnan, although the two regions have a similar climate and the same tropical rain forest at lowlands as those of southeastern Asia. The flora of the tropical region of southeastern Yunnan has a relatively high proportion of temperate east Asian characteristic families, such as Magnoliaceae, Cornaceae, Smilacaceae, Theaceae, Styracaceae, Symplocaceae, Aquifoliaceae, Caprifoliaceae and Celastraceae, while in southern Yunnan, tropical Asian characteristic families, such as Meliaceae, Annonaceae, Menispermaceae, Zingiberaceae, Apocynaceae, Asclepiadaceae, are well represented. The flora of tropical southeastern Yunnan is more closely related to eastern Asian flora, while the flora of southern Yunnan is more closely related to Indo-Malaysian flora. Based on comparisons of floristic composition and geological history between southern and tropical southeastern Yunnan, I will discuss the biogeographical divergence between these regions.

## Materials and Methods

**Figure 1:**
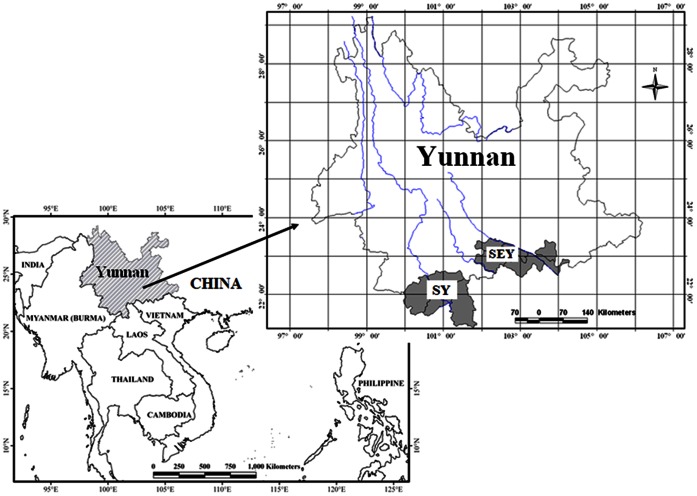
Location of study areas. SY: southern Yunnan; SEY: tropical southeastern Yunnan.

In this study, two neighboring tropical areas in southern and southeastern Yunnan were selected for comparison (Figure 1).

The tropical region of southeastern Yunnan lies between 22°26′ and 23°26′N, 104°27′ and 108°48′E, including six counties with an area of 14,389 km^2^, and the altitude varies from 75 m at the red river to 3047 m at mountain peaks. This region has a typical tropical monsoon climate at lowlands with an annual mean temperature of 22.8°C, ≥10°C annual accumulative temperature of 8246°C, and annual precipitation of 1764 mm. A total of 4996 species and 1357 genera in 186 families of seed plants have been recognized from the region [28].

Tropical southern Yunnan is delimited here to the Xishuangbanna administrative region. It lies between 21°09′ and 22°36′ N, 99°58′ and 101°50′ E, including three counties. The region comprises an area of 19690 km^2^ and lies between 480 m at the bottom of the lowest valley in the southern (Mekong River) to 2430 m at the highest peak in the north. The region has a typical tropical monsoon climate at lowlands with an annual mean temperature of 21°C, ≥10°C annual accumulative temperature of 7639°C, and annual precipitation of 1532 mm. A total of 4150 native species including subspecies and varieties from 1240 genera and 183 families of seed plants have been recognized from southern Yunnan [29].

Circumscription of families and species followed the nomenclature of w^3^TROPICOS (http://mobot.mobot.org/W3T/Search/vast.html).

Based on these plant lists, floristic and geographical attributes of these two floras were analyzed. Distribution patterns of seed plants in each region were quantified at the generic level based on Wu’s documentation [30], [31] and at the family level following Wu et al. [32]. Comparisons of both floristic composition and geographical elements were made to assess the floristic divergence between the two regions.

## Results

### Floristic Composition

A total of 4996 species and 1357 genera in 186 families of seed plants from tropical southeastern Yunnan, and a total of 4150 native species (including subspecies and varieties) from 1240 genera and 183 families of seed plants from southern Yunnan were recognized. Both floras are comprised of almost the same primary species-rich families, such as Fabaceae, Orchidaceae, Rubiaceae, Poaceae, Asteraceae, Euphorbiaceae, Lauraceae and Urticaceae. However, among the secondary species-rich families, tropical families, such as Zingiberaceae, Asclepiadaceae, Apocynaceae, Annonaceae, are more dominant in the flora of southern Yunnan, while those with a mainly temperate distribution, such as Ericaceae, Theaceae, Liliaceae and Araliaceae, are more dominant in the flora of tropical southeastern Yunnan (Table 1).

**Table 1:** The twenty families with most species richness among the floras of southern and southeastern Yunnan.

Flora of southern Yunnan			Flora of tropical southeastern Yunnan		
Family	No. sp.	% the flora	Family	No. sp.	% the flora
Orchidaceae	377	9.08	Fabaceae	285	5.7
Fabaceae	261	6.29	Orchidaceae	276	5.52
Rubiaceae	201	4.84	Rubiaceae	235	4.7
Poaceae	189	4.55	Poaceae	219	4.38
Euphorbiaceae	148	3.57	Asteraceae	180	3.6
Lamiaceae	139	3.35	Lamiaceae	151	3.02
Asteraceae	137	3.30	Lauraceae	141	2.82
Lauraceae	105	2.53	Urticaceae	134	2.68
Urticaceae	84	2.02	Euphorbiaceae	126	2.52
**Zingiberaceae**	84	2.02	Rosaceae	124	2.48
Moraceae	83	2.00	Fagaceae	109	2.18
Acanthaceae	77	1.86	Moraceae	104	2.08
**Asclepiadaceae**	66	1.59	**Ericaceae**	96	1.92
Cyperaceae	63	1.52	Cyperaceae	87	1.74
**Cucurbitaceae**	60	1.45	Acanthaceae	85	1.7
Fagaceae	60	1.45	**Theaceae**	81	1.62
Rosaceae	59	1.42	**Gesneriaceae**	79	1.58
**Annonaceae**	57	1.37	**Celastraceae**	65	1.3
**Apocynaceae**	56	1.35	**Liliaceae**	64	1.28
**Vitaceae**	56	1.35	**Araliaceae**	64	1.28

* Bold lettering indicates dominant families, which are in one of the two compared floras respectively.

### Biogeographical Divergence of the Tropical Floras of Yunnan

The floristic similarities between the flora of southeastern and southern Yunnan is up to 99% at the family level, and 80% at the generic level, but 50.7% at the specific level [28].

Although the floras of tropical southeastern and southern Yunnan have a similar composition at the family level and share many very species-rich families, they differ in secondary species-rich families. The families Aquifoliaceae, Ericaceae, Hydrangeaceae, Magnoliaceae, Rosaceae, Sapindaceae, and Theaceae have greater species richness in tropical southeastern Yunnan (Table 2) and most are dominant in the temperate east Asian flora. Additionally, there are 14 families in tropical southeastern Yunnan that are not found in southern Yunnan: Dipsacaceae, Clethraceae, Coriariaceae, Eupteleaceae, Diapensiaceae, Dipentodontaceae, Nyctaginaceae, Taxaceae, Taxodiaceae, Tetracentraceae, Hippocrateaceae, Toricelliaceae, Valerianaceae and Pentaphylacaceae; while only one family, Calophyllaceae, is restricted to southern Yunnan.

**Table 2:** Families with conspicuously more species richness in the southeastern Yunnan than the southern Yunnan.

Families	Distribution type	No. of species in southeastern Yunnan	No. of species in southern Yunnan
Rosaceae	Cosmopolitan	124	59
Ericaceae	Cosmopolitan	96	24
Theaceae	Pantropic	80	33
Magnoliaceae	East Asia and North America disjunct	53	13
Sapindaceae	Pantropic	50	22
Aquifoliaceae	Tropical Asia and Tropical America disjunct	35	13
Primulaceae	Cosmopolitan	28	12
Actinidiaceae	Tropical Asia and Tropical America disjunct	26	9
Symplocaceae	Pantropic	25	14
Cornaceae	North Temperate	22	13
Styracaceae	Tropical Asia and Tropical America disjunct	19	9
Hydrangeaceae	North Temperate	17	4
Lardizabalaceae	Tropical Asia and Tropical America disjunct	12	4
Saxifragaceae	Cosmopolitan	11	2
Betulaceae	North Temperate	11	5
Hamamelidaceae	North Temperate	11	2
Papaveracea	North Temperate	10	2
Berberidacea	North Temperate	8	2
Cycadaceae	Tropical Asia to Tropical Australia	6	2
Helwingiaceae	East Asia	5	1

Although the generic similarities between the flora of tropical southeastern and southern Yunnan is up to 80%, c. 340 tropical southeastern genera are not found in southern Yunnan, and 230 southern genera are not found in tropical southeastern Yunnan. For example, the genera *Amentotaxus, Amesiodendron, Carissa*, *Clethra, Delavaya*, *Deutzianthus, Dipterocarpus, Exbucklandia, Gonocarym, Hopea, Licuala, Lysidice, Madhuca*, *Pavieasia, Rhodoleia, Rhoiptelea,* are found only in tropical southeastern Yunnan, while *Anogeissus, Arytera, Bruinsmia, Calophyllum, Gymnanthes, Kopsia, Neuropeltis, Polyosma* are found only in southern Yunnan.

**Figure 2:**
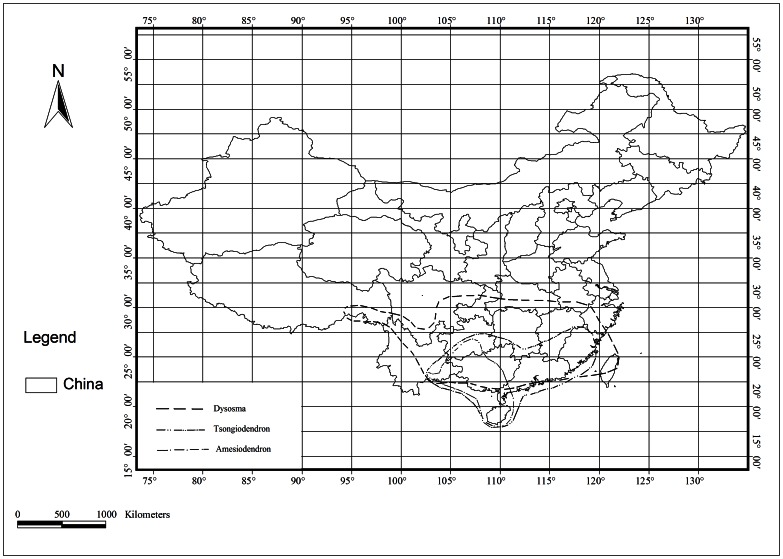
Chinese endemic genera in tropical southeastern Yunnan.

Significantly, a number of genera are found in tropical southeastern Yunnan but not in southern Yunnan: 57 genera of east Asian distribution, including *Acanthopanax, Akebia, Dysosma, Dichocarpum, Euptelea, Keteleeria, Platycarya, Trachycarpu* and *Toricellia,* 53 genera of north temperate distribution, including *Aesculus, Berberis, Coriaria, Cotoneaster, Ribes, Spiraea*, *Taxus* and *Tilia,* 22 Chinese endemic genera, including *Delavaya, Dipteronia, Emmenopterys, Excentrodendro, Ferrocalamus, Glyptostrobus, Manglietiastrum, Metapanax* and *Whytockia,* and 17 genera of east Asia and north America disjunct distribution (Table 3). Although these two neighboring regions have a similar tropical monsoon climate and similar tropical rainforests (Figure 2) there is a conspicuous divergence in these floras.

**Table 3:** Generic-level diversity respectively in the southern Yunnan and tropical southeastern Yunnan across geographical elements.

Geographical element at generic level	Genera only in southern Yunnan	%	Genera only in tropical southeastern Yunnan	%
1 Cosmopolitan	8	3.42	6	1.72
2 Pantropic	47	20.09	27	7.74
3 Tropical Asia and Tropical America disjunct	5	2.14	6	1.72
4 Old World Tropic	16	6.84	9	2.58
5 Tropical Asia to Tropical Australia	28	11.97	14	4.01
6 Tropical Asia to Tropical Africa	19	8.12	23	6.59
7 Tropical Asia	82	35.04	98	28.08
8 North Temperate	5	2.14	53	15.19
9 East Asia and North America disjunct	2	0.85	17	4.87
10 Old World Temperate	7	2.99	14	4.01
11 Temperate Asia	1	0.43	2	0.57
12 Mediterranean, W Asia to C Asia	1	0.43	1	0.29
13 C Asia	2	0.85	0	0.00
14 East Asia	7	2.99	57	16.33
15 Endemic to China	4	1.71	22	6.30
Total	234	100.00	349	100.00

## Discussion and Conclusions

Geographical elements at the generic level reveal that the floras of southern and tropical southeastern Yunnan are dominated by tropical elements, of which, the tropical Asian elements make up the highest proportion. Both floras were therefore categorized as the tropical Asian flora in the floristic regionalization of the world [1], [2], i.e. they belong to the Malaysia subkingdom of the Paleotropical kingdom. However, the families with temperate distributions, such as Rosaceae, Fagaceae, Ericaceae, Theaceae, Hamamelidaceae, Magnoliaceae, Berberidacea, Araliaceae, Aquifoliaceae, have conspicuously more species in tropical southeastern Yunnan, and 14 mainly east Asian families, such as Eupteleaceae, Dipentodontaceae, Taxaceae, Tetracentraceae and Toricelliaceae, are found in tropical southeastern Yunnan, but not in southern Yunnan. A total of 340 genera in tropical southeastern Yunnan are not found in southern Yunnan. Of these, 57 are of east Asian distribution, 53 of north temperate distribution, and 22 are Chinese endemics; revealing that the flora of tropical southeastern Yunnan has some intrinsic affinity to the flora of east Asia.

**Figure 3:**
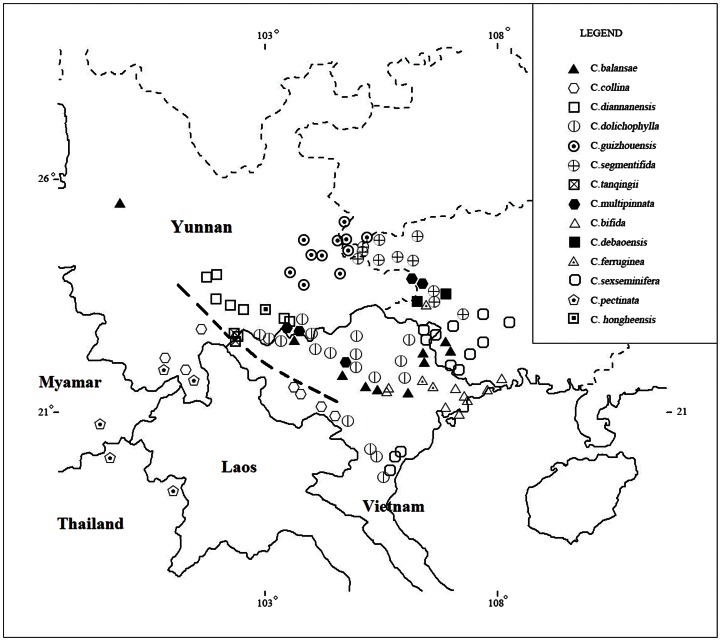
Distributions of *Cycas* species in Yunnan and neighboring areas. The dash-dot line shows a natural demarcation of *Cycas* species between southern and southeastern Yunnan.

At the specific level, a biogeographical divergence between southern and tropical southeastern Yunnan can be identified. For example, six species of the primitive seed plant *Cycas* is found in tropical southeastern Yunnan, and only two species in southern Yunnan. The two species in southern Yunnan are from sect. Stangorioides, while the six species in tropical southeastern Yunnan are from sect. Indosinenses [33], [34] (Figure 3).

Phylogeography could give implications on the divergence, evolution, and speciation of plants. References on the phylogeographic studies from the tropical areas of Yunnan have not been traced although many have been done in east Asia. Further phylogeographic studies are needed to discuss the biogeographical divergence between southern and tropical southeastern Yunnan.

The distributions of some animal taxa in Yunnan are also biogeographically divergent. Mammal species, such as the northern pig-tailed Macaque (*Macaca leonine),* Gaur *(Bos gaurus),* Banteng (*Bos javanicus),* large-toothed rat (*Dacnomys millardi*), and the Asian elephant are distributed only in southern Yunnan, while the pygmy slow loris *(Nycticebus pygmaeus),* Sun bear (*Helarctos malayanus)* and Owston’s palm civet (*Chrotogale owstoni)* are distributed in tropical southeastern Yunnan [35].

Although it is logical that the flora of tropical southeastern Yunnan with much larger altitudinal range, has more temperate families and genera than the flora of southern Yunnan, the conspicuous disparity between the two floras could not be explained only by the difference of their altitudinal ranges.

**Figure 4:**
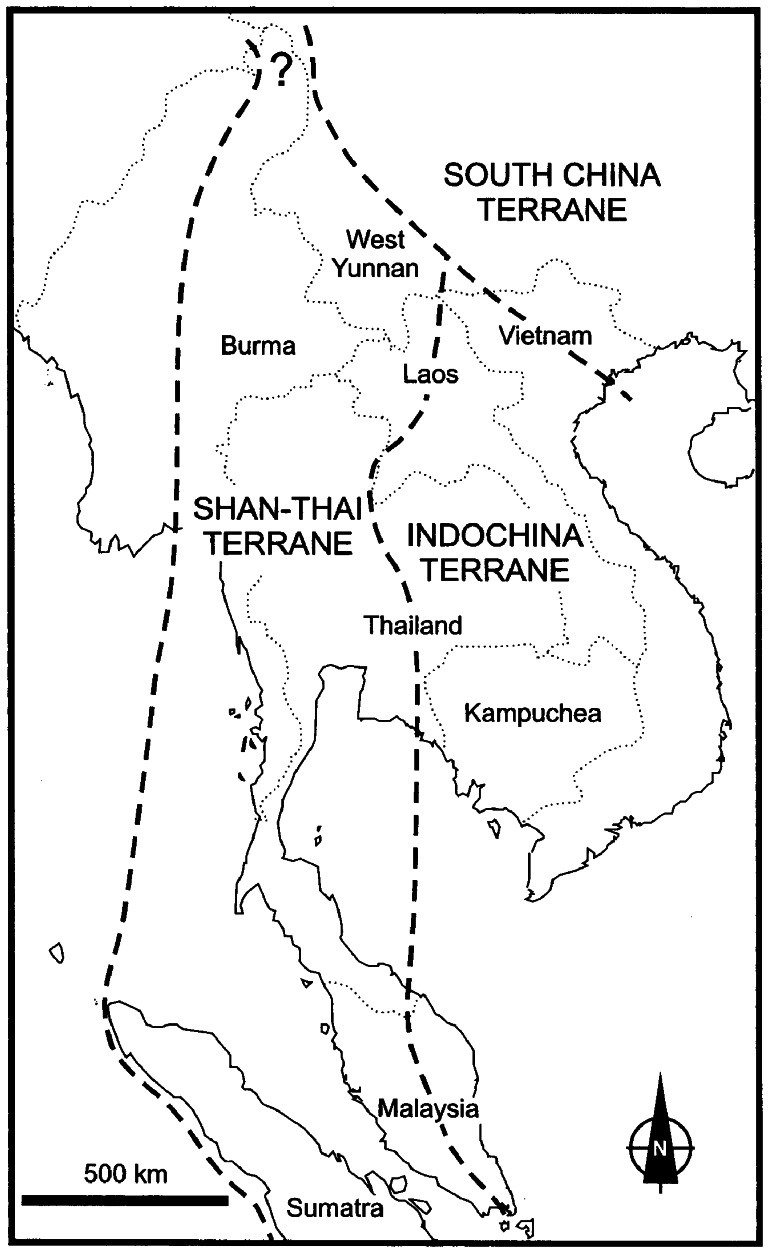
Map showing the boundaries of the various terrains in SE Asia. (from Fotey et al., 1998).

Tropical southeastern Yunnan was derived from the Southern China Geoblock or Yangzi Block, while southern Yunnan derived from Shan-Thai Geoblock or Simao Block (Figure 4) [36], [37], [38], [39], [40]. There was a deep fault between the two regions during the early Tertiary. Southern Yunnan rose completely from a maritime downfold with the uplift of Himalayas until Tertiary, while southeastern Yunnan, as part of Yangzi Block, had been a land environment since the Mesozoic [41].

**Figure 5:**
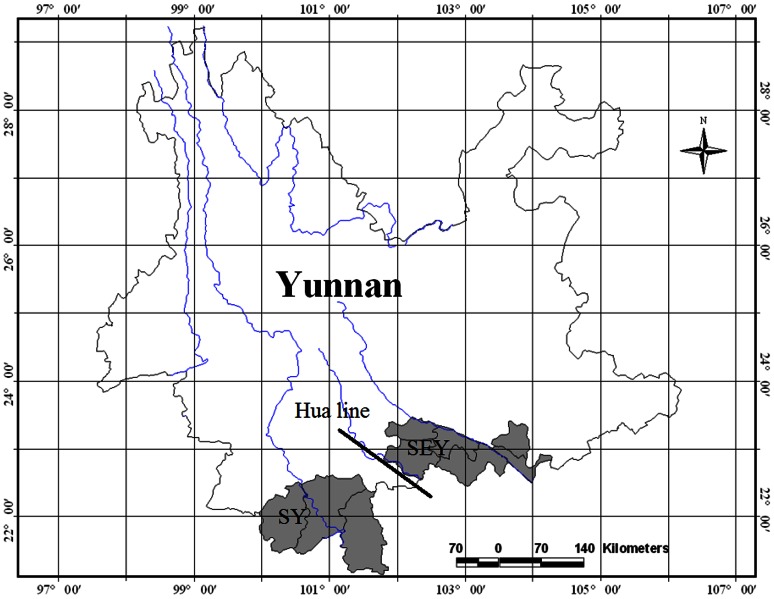
Hypothetical biogeographical line (Hua line) between southern and tropical southeastern Yunnan. Dark areas are southern Yunnan (SY), and tropical southeastern Yunnan (SEY).

Although the tropical rain forests in southern and southeastern Yunnan are phyisiognomically very similar, some dominant tropical rain forest tree species in southeastern Yunnan, such as *Saraca dives, Lysidice rhodostegia, Amesiodendron chinense, Deutzianthus tonkinensis, Zenia insignis* and *Garcinia paucinervis,* are not found in southern Yunnan. These species occur to the east of the Lixianjiang River. The Lixianjiang River also divides the *Cycas* Sections Stangorioides (southern Yunnan) and Indosinenses (southwestern Yunnan) (Figure 3) and is a natural demarcation for many other plants and animals between southern and southeastern Yunnan. It is also the geographical demarcation between the Mekong drainage area and the red river drainage area. The Lixianjiang River is, therefore, considered a hypothetical biogeographical line between tropical southern and southeastern Yunnan (Figure 5), named the “Hua line” [42]. The possible biogeographical line was further supported by using a cluster analysis of species presence/absence in Yunnan [43]. The line well matches with the geological Song Ma zone between the southern China and the Shan-Thai-Indochina Geoblock.
